# Ratio of Early Mitral Inflow Velocity to the Global Diastolic Strain Rate and Global Left Ventricular Longitudinal Systolic Strain Predict Overall Mortality and Major Adverse Cardiovascular Events in Hemodialysis Patients

**DOI:** 10.1155/2019/7512805

**Published:** 2019-09-05

**Authors:** Jiun-Chi Huang, Ho-Ming Su, Pei-Yu Wu, Jia-Jung Lee, Wen-Hsien Lee, Szu-Chia Chen, Yi-Wen Chiu, Ya-Ling Hsu, Jer-Ming Chang, Hung-Chun Chen

**Affiliations:** ^1^Graduate Institute of Clinical Medicine, College of Medicine, Kaohsiung Medical University, Kaohsiung, Taiwan; ^2^Division of Nephrology, Department of Internal Medicine, Kaohsiung Medical University Hospital, Kaohsiung Medical University, Kaohsiung, Taiwan; ^3^Department of Internal Medicine, Kaohsiung Municipal Hsiao-Kang Hospital, Kaohsiung Medical University, Kaohsiung, Taiwan; ^4^Faculty of Medicine, College of Medicine, Kaohsiung Medical University, Kaohsiung, Taiwan; ^5^Division of Cardiology, Department of Internal Medicine, Kaohsiung Medical University Hospital, Kaohsiung Medical University, Kaohsiung, Taiwan; ^6^Faculty of Renal Care, College of Medicine, Kaohsiung Medical University, Kaohsiung, Taiwan; ^7^Graduate Institute of Medicine, College of Medicine, Kaohsiung Medical University, Kaohsiung, Taiwan; ^8^Department of Internal Medicine, Kaohsiung Municipal Cijin Hospital, Kaohsiung Medical University, Kaohsiung, Taiwan

## Abstract

**Background:**

The ratio of early mitral inflow velocity to the global diastolic strain rate (E/E'sr) and global longitudinal systolic strain (GLS) of the left ventricle (LV) are emerging indices of diastolic and systolic functions, respectively, for the LV. Their prognostic significance in the prediction of mortality and cardiovascular (CV) outcomes remains underexplored in hemodialysis (HD) patients.

**Methods:**

This prospective study included 190 maintenance HD patients. The E/E'sr ratio and GLS were assessed using two-dimensional speckle tracking echocardiography. The clinical outcomes included overall mortality, CV mortality, and major adverse cardiovascular events (MACE). The associations between the E/E'sr ratio, GLS, and clinical outcomes were evaluated using multivariate Cox regression analysis. The incremental values of the E/E'sr ratio and GLS in outcome prediction were assessed by *χ*^2^ changes in Cox models.

**Results:**

Over a median follow-up period of 3.7 years, there were 35 overall deaths, 16 CV deaths, and 45 MACE. Impaired diastolic function with a higher E/E'sr ratio was associated with overall mortality (HR, 1.484; 95% CI, 1.201−1.834; *p* < 0.001), CV mortality (HR, 1.584; 95% CI, 1.058–2.371; *p* = 0.025), and MACE (HR, 1.205; 95% CI, 1.040−1.397; *p* = 0.013) in multivariate adjusted Cox analysis. Worsening GLS was associated with overall mortality (HR, 1.276; 95% CI, 1.101−1.480; *p* = 0.001), CV mortality (HR, 1.513; 95% CI, 1.088−2.104; *p* = 0.014), and MACE (HR, 1.214; 95% CI, 1.103−1.337; *p* < 0.001). The E/E'sr ratio and GLS had better outcome prediction than the E to early diastolic mitral annular velocity (E/E') ratio and left ventricular ejection fraction (LVEF). Moreover, adding the E/E'sr ratio and GLS to Cox models containing relevant clinical and conventional echocardiographic parameters improved the prediction of overall mortality (*p* < 0.001), CV mortality (*p* < 0.001), and MACE (*p* < 0.001).

**Conclusion:**

The E/E'sr ratio and GLS, as emerging indices of LV diastolic and systolic functions, significantly predict mortality and CV outcomes and outperform conventional echocardiographic parameters in outcome prediction in HD patients.

## 1. Introduction

Cardiovascular (CV) disease is the leading cause of mortality in patients undergoing hemodialysis (HD) [1]. Higher prevalence of traditional risk factors and functional abnormalities of the heart may contribute to this high CV risk in end-stage renal disease (ESRD) [2, 3]. Pressure and volume overload could cause such cardiac abnormalities [4, 5]. Two-dimensional (2D) speckle tracking echocardiography (STE) allows for angle-independent quantification of myocardial deformation to more accurately reflect systolic and diastolic performances of all myocardial segments [6, 7].

2D STE can assess the left ventricular (LV) early global diastolic strain rate (E'sr). Furthermore, the early mitral inflow velocity (E) to E'sr ratio has been reported to be an emerging index of LV diastolic function [8, 9] and is strongly correlated with invasively measured LV filling pressure [10–12]. The E/E'sr ratio is associated with unfavorable outcomes among patients with acute myocardial infarction [13] and systolic heart failure [14]. Global LV longitudinal systolic strain (GLS) has been recognized as a proper indicator of LV systolic function [7, 15]. Less negative GLS is associated with an increased risk of death in patients undergoing dialysis [16, 17]. However, the associations between the E/E'sr ratio and the risk of mortality and CV outcomes have never been investigated in chronic HD patients. Therefore, this study is aimed at examining the prognostic significance of the E/E'sr ratio and GLS in the prediction of overall mortality, CV mortality, and major adverse cardiovascular events (MACE) in maintenance HD patients. We further explored whether the emerging indices of LV diastolic and systolic functions outperformed the conventional echocardiographic parameters in the prediction of mortality and CV outcomes.

## 2. Materials and Methods

### 2.1. Study Patients

**Figure 1:**
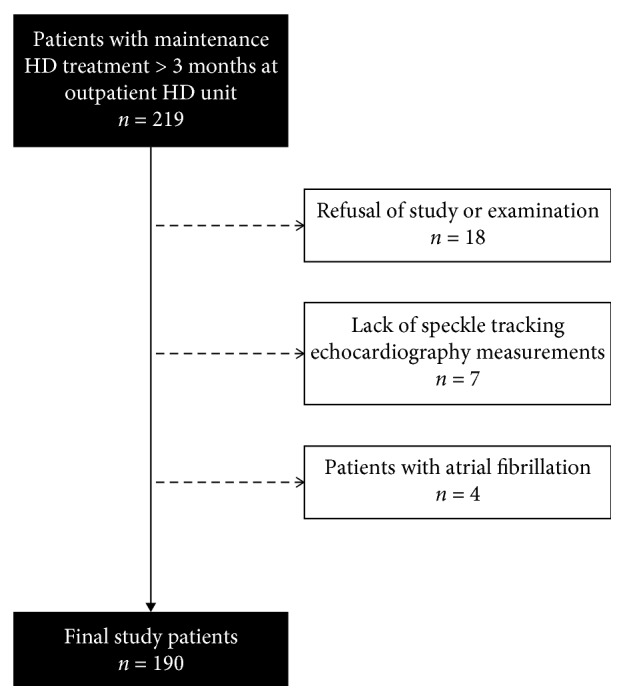
Flowchart of participants analyzed in this study.

The inclusion criterion of the present study was patients with maintenance hemodialysis (HD) > 3 months at the outpatient HD unit. This study enrolled 219 maintenance HD patients at a regional hospital in Taiwan from March to October 2014. Patients with refusal of examinations (*n* = 18), lack of STE measurements (*n* = 7), and atrial fibrillation (*n* = 4) were excluded. No patient was excluded because of poor echogenicity. Overall, 190 study patients were included (Figure 1). The study adhered to the Declaration of Helsinki and was approved by the Institutional Review Board of Kaohsiung Medical University Hospital, and all participants provided their written informed consent.

### 2.2. Echocardiographic Measurements

Patients received echocardiographic measurements in the left decubitus position by one well-experienced cardiologist, using a Vivid 7 system (GE Vingmed Ultrasound AS, Horten, Norway). The cardiologist was blind to patients' clinical information. Early diastolic velocities (E') of lateral and septal mitral annuli were averaged using Doppler tissue imaging to calculate the E/E' ratio. The LV ejection fraction (LVEF) and LV mass were calculated using the biplane Simpson's and Devereux's methods, respectively [6]. The LV mass index (LVMI) was calculated as LV mass divided by the body surface area. Left atrial volume was calculated using the biplane area-length method. The left atrial volume index (LAVI) was defined as left atrial volume divided by the body surface area. Relative wall thickness was calculated by (2 × posterior wall thickness in diastole)/LV diastolic diameter. All volumetric measurements and analyses were performed in accordance with EAE/ASE recommendations [6].

**Figure 2:**
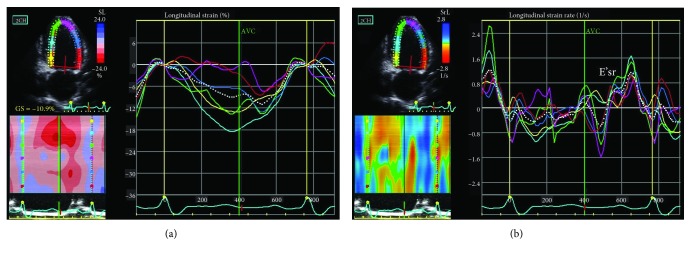
A representative example of measurements of global longitudinal strain (a) and the early global diastolic strain rate (b) from the curves of longitudinal strain and the strain rate of six segments of the left ventricle in the apical two-chamber view. AVC: aortic valve closure; E'sr: early global diastolic strain rate.

LV apical two- and four-chamber and long-axis views were obtained. The endocardial border was defined manually, and epicardial surface tracing was automatically performed by the system to create a region of interest [18]. The LV chamber was divided into six segments, with their strain and strain rate curves being analyzed. The peak segmental longitudinal systolic strain and early diastolic strain rates were determined from these curves (Figure 2). The E'sr and GLS were assessed and averaged in 18 LV segments from the three standard apical views (four-, two-, and three-chamber views). All the 18 speckle tracking segments were kept into the analysis in all patients. LV dimensions, LVEF, LAVI, LVMI, E'sr, and GLS were measured from the index beat [9, 19, 20]. A single beat was analyzed each time, and the values from three cardiac cycles were average to obtain each index. All STE data were recorded and analyzed offline using EchoPAC version 08.

### 2.3. Assessment of the Ankle-Brachial Index (ABI) and Brachial-Ankle Pulse Wave Velocity (baPWV)

ABI and baPWV were measured 10−30 minutes before the HD session using an ABI-form analyzer (Colin VP1000, Komaki, Japan) which simultaneously measured blood pressure in both arms and ankles. ABI was calculated as systolic blood pressure of the ankles divided by systolic blood pressure of the arms. The baPWV was automatically calculated as the transmission distance divided by the transmission time. The ABI is a simple and noninvasive test for establishing the diagnosis of peripheral artery disease (PAD) and a marker of generalized atherosclerosis, which are prevalent among HD patients and associated with worse clinical outcomes [21]. PAD was defined as an ABI < 0.95 [22, 23].

### 2.4. Evaluation of Aortic Arch Calcification (AoAC) and the Cardiothoracic Ratio by Chest X-Rays

An experienced radiologist blind to the patients' clinical information reviewed their chest X-rays and assessed AoAC using the scale proposed by Ogawa et al. [24]. The aortic arch was divided into 16 sections on the chest X-rays, and the number of sections with calcification was counted. The cardiothoracic ratio, assessed by the radiologist, was defined as the ratio of the transverse diameter of the cardiac shadow to the transverse diameter of the chest on the chest X-rays.

### 2.5. Demographic, Medical, and Laboratory Data

Demographic and medical data including age, gender, and comorbidities were obtained from patients' medical records and interviews. Laboratory tests were conducted using overnight fasting blood samples obtained within 1 month of enrollment. Information of the use of medications, including angiotensin-converting enzyme (ACE) inhibitors, angiotensin II receptor blockers (ARBs), *β*-blockers, and statins, was obtained from medical records.

### 2.6. Definition of Overall Mortality, CV Mortality, and MACE

All study patients' medical records and hospital courses were reviewed by two cardiologists to define the cause of death and MACE. CV mortality was defined as sudden cardiac death, fatal myocardial infarction, ventricular arrhythmia, fatal stroke, and heart failure. MACE was defined as follows: hospitalization for unstable angina, nonfatal myocardial infarction, sustained ventricular arrhythmia, hospitalization for congestive heart failure, transient ischemia attack or stroke, hospitalization for peripheral artery occlusive disease, and CV death [25]. If the patients experienced more than one MACE, only the first was analyzed. The model for MACE was censored at the development of MACE or the end of follow-up. All patients were followed until December 31, 2017, or the study endpoint (overall or CV mortality).

### 2.7. Statistical Analysis

All statistical analyses were carried out using SPSS version 19.0 (SPSS Inc., Chicago, IL, USA) for Windows. Data are expressed as percentages, mean ± standard deviation, or median (25^th^–75^th^ percentile) for the dialysis vintage, triglycerides, high-sensitivity C-reactive protein (hs-CRP), and AoAC. The study patients were stratified into three groups according to tertiles of the E/E'sr ratio, with the 1^st^ tertile as the reference category. Multiple comparisons among the study groups were performed using one-way analysis of variance followed by the post hoc Bonferroni test. These study patients were also classified into two groups according to a preserved GLS (≤−16%) or impaired GLS (>−16%) [26, 27]. Differences between groups were analyzed using the chi-square test for categorical variables and the independent *t*-test for continuous variables. Cox proportional hazards analysis was performed to evaluate the associations between the E/E'sr ratio and GLS and development of overall mortality, CV mortality, or MACE. The adjusted covariates included age, sex, dialysis vintage, diabetes mellitus (DM), coronary artery disease, cerebrovascular disease, body mass index, systolic blood pressure, ABI < 0.95, baPWV, the cardiothoracic ratio, AoAC, albumin, triglycerides, total cholesterol, hemoglobin, creatinine, calcium-phosphorous product, hs-CRP, and the use of ACE inhibitors or ARBs, *β*-blockers, and statins. Survival curves for cumulative probability of overall mortality, CV mortality and MACE were illustrated using the Kaplan-Meier method and compared among mentioned groups of patients by the log-rank test. Direct comparisons between the E/E' ratio and the E/E'sr ratio, as well as GLS and LVEF, were performed using multivariate models and assessed by changes in *χ*^2^. Incremental model performance of the E/E'sr ratio and GLS over clinical and echocardiographic variables was assessed by comparing the model *χ*^2^ at each step. A *p* value<0.05 was considered statistically significant.

## 3. Results

A total of 190 study patients were included. The mean age was 60.7 ± 11.7 years, and there were 98 males and 92 females. The mean values of the E/E'sr ratio and GLS were 64.5 ± 25.5 cm and −16.8 ± 4.1%, respectively. The patients were stratified into three groups according to tertiles of the E/E'sr ratio (<50.4 cm, 50.4–67.9 cm, and >67.9 cm). Patients belonging to the 3^rd^ tertile of the E/E'sr ratio had higher prevalence of DM and coronary artery disease, higher cardiothoracic ratio, higher AoAC, lower serum creatinine, higher prevalence of ACE inhibitors or ARB and *β*-blocker use, higher LAVI, higher LVMI, lower LVEF, higher E/E' ratio, higher E/A ratio, lower E' wave, lower E'sr, and less negative GLS compared to patients in the 1^st^ tertile of the E/E'sr ratio (Table 1).

Table 2 shows the comparison of baseline characteristics between patients with preserved GLS (≤−16%) or impaired GLS (>−16%). Compared to patients with preserved GLS, those with impaired GLS had higher prevalence of DM, coronary artery disease, and cerebrovascular disease; higher systolic blood pressure; higher prevalence of ABI < 0.95; higher AoAC; higher hemoglobin; higher calcium-phosphorous product; higher LVMI; lower LVEF; lower E' wave; lower E'sr; and higher E/E'sr ratio.

**Figure 3:**
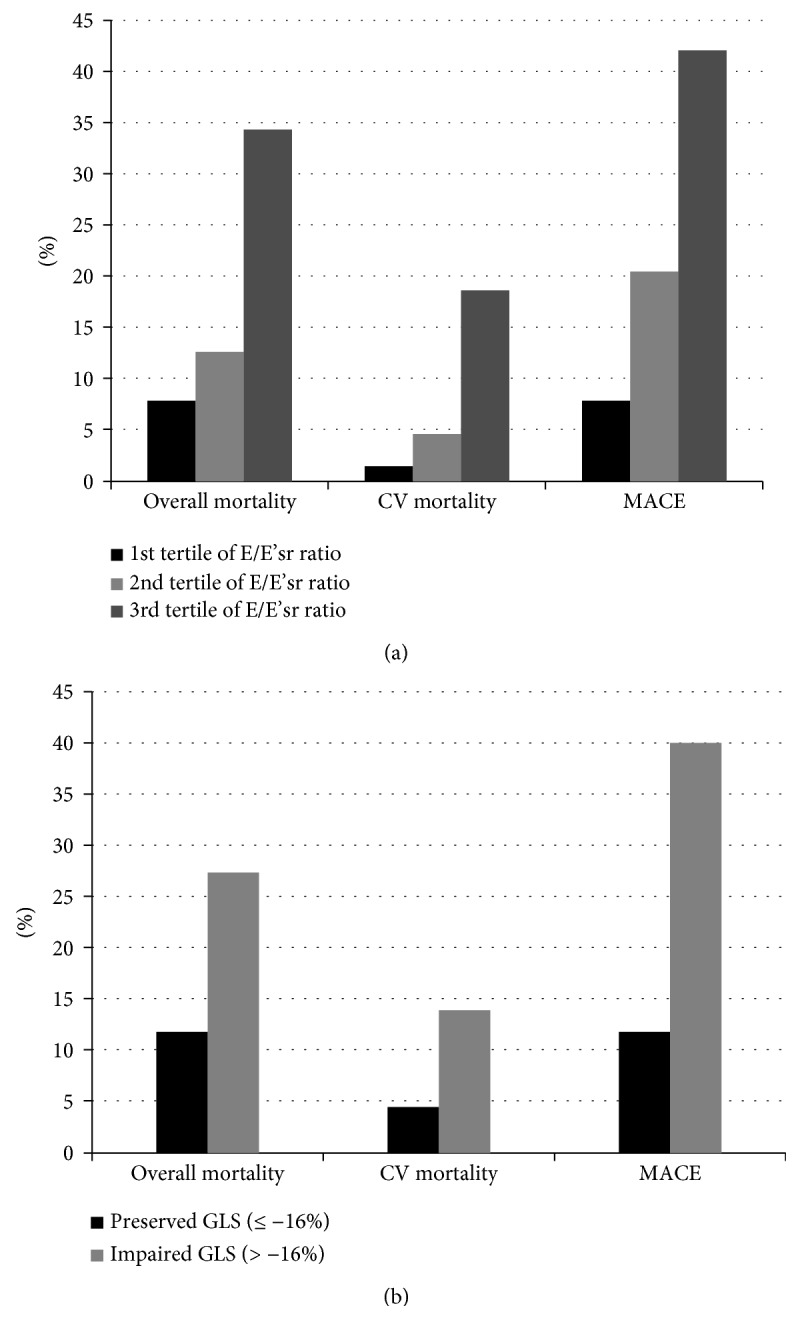
Incidence rates of overall mortality, CV mortality, and MACE over a median of 3.7 years among patients stratified by E/E'sr tertiles (a) and between patients with preserved GLS (≤−16%) or impaired GLS (>−16%) (b).

As shown in Figure 3(a), the cumulative incidence rates of overall mortality, CV mortality, and MACE were highest among patients in the 3^rd^ tertile of the E/E'sr ratio, intermediate among those in the 2^nd^ tertile and lowest among those in the 1^st^ tertile (*p* < 0.001 for the trend). The incidence rates of overall mortality (*p* = 0.006), CV mortality (*p* = 0.024), and MACE (*p* < 0.001) were higher among patients with impaired GLS in comparison with those with preserved GLS (Figure 3(b)).

### 3.1. Risk of Overall Mortality

**Figure 4:**
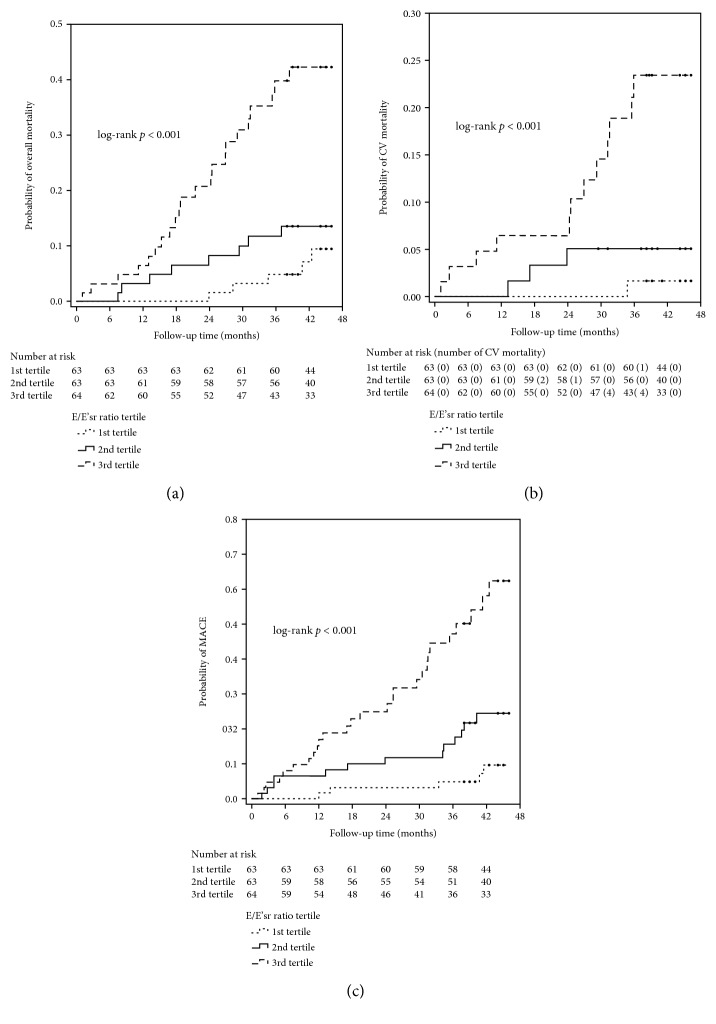
Kaplan-Meier curves for cumulative probability of overall mortality (log-rank *p* < 0.001) (a), CV mortality (log-rank *p* < 0.001) (b), and MACE (log-rank *p* < 0.001) (c) among patients stratified by E/E'sr tertiles.

**Figure 5:**
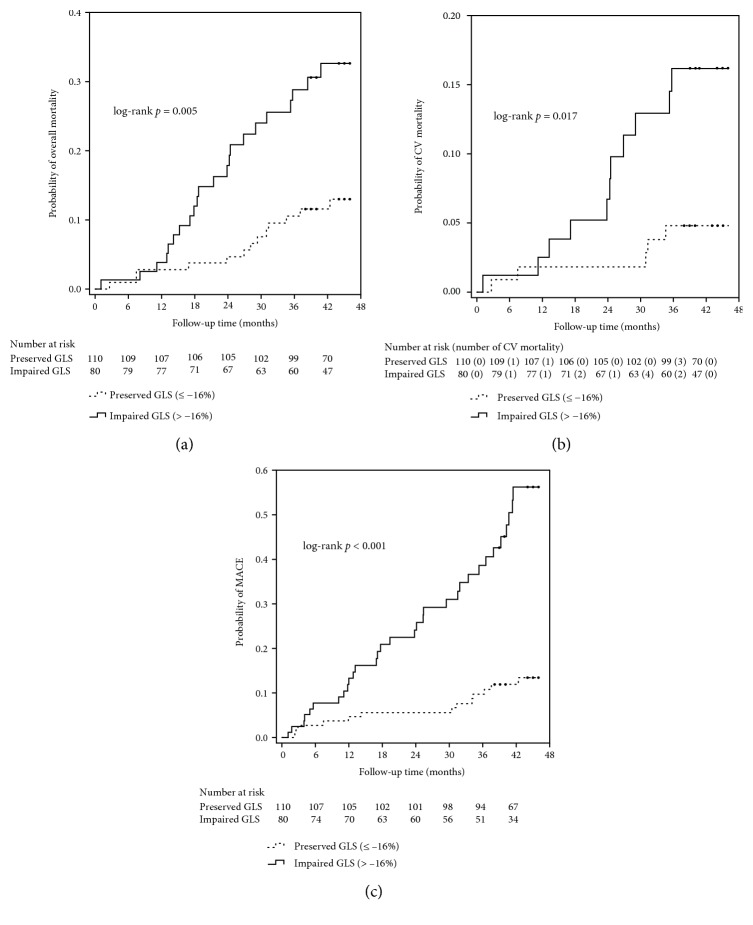
Kaplan-Meier curves for cumulative probability of overall mortality (log-rank *p* = 0.005) (a), CV mortality (log-rank *p* = 0.017) (b), and MACE (log-rank *p* < 0.001) (c) in patients with preserved GLS (≤−16%) or impaired GLS (>−16%).

Over a median follow-up of 3.7 years (interquartile range: 3.3-3.8 years), there were 35 (18.4%) deaths, including fatal CV events (*n* = 16), sepsis or septic shock (*n* = 15), gastrointestinal bleeding (*n* = 2), malignancy (*n* = 1), and liver failure (*n* = 1) among study patients. The Kaplan-Meier curves for the cumulative probability of overall mortality according to tertiles of the E/E'sr ratio (Figure 4(a)) show substantially higher probability of overall mortality among patients in the 3^rd^ tertile of the E/E'sr ratio compared to patients in the 1^st^ or 2^nd^ tertile of the E/E'sr ratio (*p* < 0.001 by the log-rank test). As shown in Figure 5(a), patients with impaired GLS had higher probability of overall mortality compared to those with preserved GLS (*p* = 0.005 by the log-rank test).

Table 3 displays the hazard ratios (HR) of the E/E'sr ratio and GLS for overall mortality with and without adjustment for demographic, clinical, biochemical factors. A high E/E'sr ratio (per 10 cm) was significantly associated with overall mortality in the unadjusted model (HR, 1.191; 95% confidence interval (CI), 1.090−1.301; *p* < 0.001), in the age- and sex-adjusted model (HR, 1.185; 95% CI, 1.074−1.308; *p* = 0.001), and in the multivariable model adjusted for age, sex, dialysis vintage, DM, coronary artery disease, cerebrovascular disease, BMI, systolic blood pressure, ABI < 0.95, baPWV, cardiothoracic radio, AoAC, albumin, triglycerides, total cholesterol, hemoglobin, creatinine, calcium-phosphorous product, and hs-CRP. This association holds significant (HR, 1.484; 95% CI, 1.201−1.834; *p* < 0.001) after being further adjusted for medication use of ACE inhibitors or ARBs, *β*-blockers, and statins.

Furthermore, GLS (per 1%) was significantly associated with overall mortality in the unadjusted model (HR, 1.127; 95% CI, 1.043−1.219; *p* = 0.003), in the age- and sex-adjusted model (HR, 1.139; 95% CI, 1.046−1.239; *p* = 0.003), and in the full multivariable adjusted model (HR, 1.276; 95% CI, 1.101−1.480; *p* = 0.001).

### 3.2. Risk of CV Mortality

Sixteen (8.4%) CV deaths were recorded during the follow-up period, including sudden cardiac death (*n* = 8), myocardial infarction (*n* = 4), ventricular arrhythmia (*n* = 1), fatal stroke (*n* = 2), and heart failure (*n* = 1). The Kaplan-Meier curves (Figure 4(b)) show higher cumulative probability of CV mortality among patients in the 3^rd^ tertile of the E/E'sr ratio compared to patients in the 1^st^ or 2^nd^ tertile of the E/E'sr ratio (*p* < 0.001 by the log-rank test).  Figure 5(b) shows that compared to patients with preserved GLS, those with impaired GLS had higher cumulative probability of CV mortality (*p* = 0.017 by the log-rank test).

As shown in Table 3, a high E/E'sr ratio (per 10 cm) was associated with CV mortality in the unadjusted model (HR, 1.271; 95% CI, 1.135−1.423; *p* < 0.001), in the age- and sex-adjusted model (HR, 1.261; 95% CI, 1.112−1.429; *p* < 0.001), and in the full multivariable adjusted model (HR, 1.584; 95% CI, 1.058−2.371; *p* = 0.025). GLS (per 1%) was associated with CV mortality in the unadjusted model (HR, 1.202; 95% CI, 1.075−1.343; *p* = 0.001), in the age- and sex-adjusted model (HR, 1.208; 95% CI, 1.072−1.360; *p* = 0.002), and in the full multivariable adjusted model (HR, 1.513; 95% CI, 1.088−2.104; *p* = 0.014).

### 3.3. Risk of MACE

Forty-five (23.7%) MACE were documented during the follow-up period, including hospitalization for heart failure (*n* = 5), coronary artery disease (*n* = 12), ventricular arrhythmia (*n* = 3), stroke (*n* = 3), peripheral artery disease (*n* = 6), and CV deaths (*n* = 16). As shown in Figures 4(c) and 5(c), patients in the 3^rd^ tertile of the E/E'sr ratio had higher cumulative probability of MACE compared to patients in the 1^st^ or 2^nd^ tertile of the E/E'sr ratio (*p* < 0.001 by the log-rank test) and higher cumulative probability of MACE among patients with impaired GLS compared to those with preserved GLS over the follow-up period (*p* < 0.001 by the log-rank test).

Table 3 shows that the high E/E'sr ratio (per 10 cm) was associated with MACE in the unadjusted model (HR, 1.188; 95% CI, 1.101−1.282; *p* < 0.001) and in the full multivariable adjusted model (HR, 1.205; 95% CI, 1.040−1.397; *p* = 0.013). GLS (per 1%) was associated with MACE in the unadjusted model (HR, 1.174; 95% CI, 1.100−1.254; *p* < 0.001) and in the full multivariable adjusted model (HR, 1.214; 95% CI, 1.103−1.337; *p* < 0.001).

### 3.4. Comparison of the E/E' Ratio and the E/E'sr Ratio to Overall Mortality, CV Mortality, and MACE

As shown in Table 4, the addition of the E/E' ratio to the basic model (comprises age, sex, dialysis vintage, DM, coronary artery disease, cerebrovascular disease, BMI, systolic blood pressure, ABI < 0.95, baPWV, cardiothoracic ratio, AoAC, album, triglycerides, total cholesterol, hemoglobin, creatinine, calcium-phosphorous product, hs-CRP, and the use of ACE inhibitors or ARBs, *β*-blockers, and statins) did not significantly improve the prediction for overall mortality, CV mortality, and MACE. In contrast, the addition of the E/E'sr ratio to the basic model showed significant improvement of the prediction for overall mortality (*χ*^2^ change = 13.914, *p* < 0.001), CV mortality (*χ*^2^ change = 6.833, *p* = 0.009), and MACE (*χ*^2^ change = 5.424, *p* = 0.020).

### 3.5. Comparison of LVEF and GLS to Overall Mortality, CV Mortality, and MACE

As shown by a direct comparison in Table 4, the addition of LVEF to the basic model did not significantly improve the outcome prediction. However, the addition of GLS to the basic model showed significant improvement of the prediction for overall mortality (*χ*^2^ change = 12.007, *p* = 0.001), CV mortality (*χ*^2^ change = 10.189, *p* = 0.001), and MACE (*χ*^2^ change = 15.682, *p* < 0.001).

### 3.6. Incremental Value of the E/E'sr Ratio and GLS in Relation to Overall Mortality, CV Mortality, and MACE

**Figure 6:**
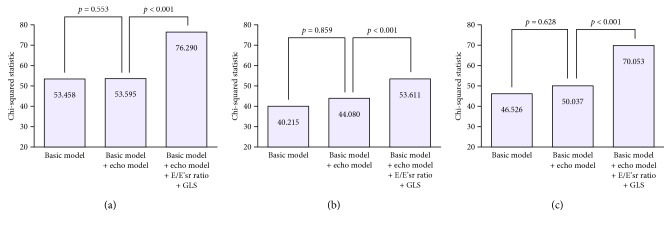
Addition of the E/E'sr ratio and GLS to the basic model and echo model improved the prediction of overall mortality (a), CV mortality (b), and MACE (c). Model *χ*^2^ values are presented for a series of Cox models.

The incremental values of the E/E'sr ratio and GLS in the prediction of overall mortality, CV mortality, and MACE are shown in Figures 6(a)–6(c), respectively. The addition of the echo model (comprises LAVI, LVMI, LVEF, and the E/E' ratio) to the basic model did not result in a significant improvement in the prediction of adverse outcomes. Moreover, the addition of the E/E'sr ratio and GLS to the basic model plus the echo model resulted in a further significant improvement in the prediction of overall mortality (*p* < 0.001), CV mortality (*p* < 0.001), and MACE (*p* < 0.001).

## 4. Discussion

In this study, we found that the higher E/E'sr ratio and GLS were independently associated with increased risk of overall mortality, CV mortality, and MACE in HD patients. The E/E'sr ratio was better than the E/E' ratio, and GLS was better than LVEF in predicting adverse outcomes. Furthermore, the E/E'sr ratio and GLS had significant incremental prognostic values beyond clinical and conventional echocardiographic parameters.

An important finding of this study highlights that the E/E'sr ratio is a novel risk factor for overall mortality, CV mortality, and MACE in patients undergoing HD. At present, the E/E' ratio is a recommended modality to assess LV diastolic function [8, 28] and associated with mortality in HD patients [29] but it still has some drawbacks such as angle dependency and risk of errors with angulations > 20°. The E'sr obtained by STE from the whole left ventricle could overcome these limitations and more accurately represent global LV relaxation. Thus, the E/E'sr ratio correlates the LV filling pressures better than the E/E' ratio does [10, 11]. The deformation-based E/E'sr ratio provides more important information with regard to global myocardial relaxation than the velocity-based E/E' ratio, and the E/E'sr ratio was independently associated with adverse outcomes in several disease states and the general population [14, 30–32]. Furthermore, we found the superiority of the E/E'sr ratio over the E/E' ratio in predicting mortality and CV outcomes and the incremental prognostic value of the E/E'sr ratio and GLS over the conventional echocardiographic parameters in HD patients. The E'sr angle independently detects subtle myocardial motion, and it more precisely reflects LV global diastolic function compared with the E/E' ratio [32].

Evaluating LV systolic function is fundamental on echocardiography [6], and LVEF remains the most widely utilized indicator. Technical limitations in the measurement of LVEF include suboptimal endocardial definition and the formulas that make assumptions with regard to the geometry of the left ventricle [33]. Furthermore, LVEF as a measure of contractility is affected by load dependency and LVEF is insensitive to identify the subtle degree of systolic dysfunction in patients with LVEF > 45% [34]. As a result, the association between LVEF and mortality was inconsistent in certain studies [34, 35]. Our study indicates the independent effect of GLS on overall mortality, CV mortality, and MACE, and GLS outperformed LVEF in predicting unfavorable outcomes in chronic HD patients. These findings are in line with previous studies on nondialyzed CKD and on ESRD patients [16, 17, 27, 36]. GLS can assess the function of longitudinally orientated myofibers, which are most vulnerable because of their subendocardial location. Although GLS is load dependent, GLS is sensitive to detect early subendocardial changes with better reproducibility than LVEF and reflect the extent of myocardial fibrosis and uremic cardiomyopathy, even in those with preserved LVEF [16, 17, 37].

Another important finding is that the addition of the E/E'sr ratio and GLS to models containing markers of atherosclerosis and conventional indicators for LV systolic and diastolic functions as risk factors of mortality in HD patients [2, 21, 38, 39] offered incremental value in the prediction of adverse outcomes. The speckle tracking imaging is based on frame-by-frame tracking of the displacement of speckles within the myocardium during the cardiac cycle and subsequent measurement of LV deformations. This technique makes it independent on imaging factors including reverberation artifacts and attenuation. Thus, the E/E'sr ratio and GLS may be more representative of global LV function. Therefore, we suggest that the E/E'sr ratio and GLS should be measured during echocardiographic examinations to provide important prognostic information for chronic HD patients.

There are several limitations in the present study. First, the number of study patients is relatively small and the observation period may be not long enough. Second, the echocardiographic parameters were measured from the index beat. This method has been proved to be as accurate as the time-consuming method of averaging echocardiographic parameters from multiple cardiac cycles [9, 19]. Third, 2D STE generates longitudinal, radial, and circumferential deformation measurements and LV twist [40]. However, only E'sr and GLS were measured and analyzed in this study. The comparisons between these parameters with radial and circumferential strains and LV twist in predicting outcomes are warranted in the future study.

## 5. Conclusion

The E/E'sr ratio and GLS, as emerging indices of LV diastolic and systolic functions obtained from 2D STE, are useful parameters and are superior to the E/E' ratio and LVEF in the prediction of mortality and CV outcomes in maintenance HD patients and may offer an incremental value of prognostic significance over relevant clinical and conventional echocardiographic parameters.

## Figures and Tables

**Table 1:** Comparison of baseline characteristics among patients according to tertiles of the E/E'sr ratio.

Characteristics	1^st^ tertile of E/E'sr ratio (<50.4 cm) (*n* = 63)	2^nd^ tertile of E/E'sr ratio (50.4–67.9 cm) (*n* = 63)	3^rd^ tertile of E/E'sr ratio (>67.9 cm) (*n* = 64)	*p*
Age (year)	58.6 ± 10.6	60.6 ± 13.3	63.2 ± 11.0	0.083
Male gender (%)	49.2	47.6	57.8	0.465
Dialysis vintage (year)	7.6 (3.7–12.5)	6.6 (2.9–13.0)	6.3 (1.9–9.7)	0.346
Diabetes mellitus (%)	31.7	39.7	65.6^∗^^†^	<0.001
Coronary artery disease (%)	3.2	11.1	28.1^∗^^†^	<0.001
Cerebrovascular disease (%)	9.5	7.9	10.9	0.846
Body mass index (kg/m^2^)	23.1 ± 3.1	23.8 ± 4.7	24.1 ± 3.6	0.344
Systolic blood pressure (mmHg)	150.3 ± 29.9	157.4 ± 28.7	159.3 ± 23.6	0.203
Heart rate (beat/min)	79.3 ± 11.3	79.8 ± 11.0	75.7 ± 11.1	0.075
ABI < 0.95 (%)	25.4	31.7	40.6	0.105
baPWV (cm/s)	1792.9 ± 533.9	2056.0 ± 546.4^∗^	1921.7 ± 516.2	0.037
Cardiothoracic ratio (%)	48.1 ± 5.7	49.4 ± 5.9	51.5 ± 6.5^∗^	0.007
AoAC	0 (0–3.8)	3 (0–7)	3 (0-7)^∗^	0.003
Laboratory parameters				
Albumin (g/dL)	3.8 ± 0.3	3.8 ± 0.3	3.9 ± 0.3	0.846
Triglycerides (mg/dL)	125.0 (81–209)	111.0 (82–199)	142.5 (95.3–225.3)	0.647
Total cholesterol (mg/dL)	184.5 ± 39.2	172.7 ± 35.2	176.6 ± 45.1	0.247
Hemoglobin (g/dL)	10.4 ± 1.0	10.5 ± 1.3	10.6 ± 1.4	0.640
Creatinine (mg/dL)	9.9 ± 2.0	9.9 ± 2.5	8.9 ± 2.3^∗^^†^	0.020
Calcium-phosphorous product (mg^2^/dL^2^)	40.1 ± 10.2	40.8 ± 12.7	44.5 ± 11.5	0.077
hs-CRP (mg/L)	1.9 (0.8–5.4)	2.9 (0.9–6.6)	3.0 (1.0–7.2)	0.554
Medications				
ACE inhibitors or ARBs (%)	11.1	15.9	39.1^∗^^†^	<0.001
*β*-Blockers (%)	9.5	17.5	37.5^∗^^†^	<0.001
Statins (%)	20.6	19.0	26.6	0.560
Echocardiographic data				
LAVI (mL/m^2^)	27.9 ± 8.6	33.2 ± 10.6^∗^	39.1 ± 12.7^∗^^†^	<0.001
LVMI (g/m^2^)	113.2 ± 31.1	136.1 ± 33.4^∗^	157.6 ± 48.3^∗^^†^	<0.001
LVEF (%)	68.2 ± 8.7	68.0 ± 8.0	63.8 ± 12.3^∗^	0.019
Relative wall thickness	0.42 ± 0.09	0.43 ± 0.09	0.41 ± 0.12	0.607
E/E' ratio	9.3 ± 3.1	12.6 ± 5.1	21.2 ± 12.6^∗^^†^	<0.001
E/A ratio	0.74 ± 0.20	0.84 ± 0.26	1.00 ± 0.44^∗^^†^	<0.001
Deceleration time (ms)	179.9 ± 65.2	188.4 ± 51.7	194.9 ± 67.3	0.393
E' wave (cm/s)	7.3 ± 2.0	7.1 ± 2.5	5.6 ± 2.0^∗^^†^	<0.001
E'sr (1/s)	1.5 ± 0.4	1.4 ± 0.3^∗^	1.1 ± 0.3^∗^^†^	<0.001
E/E'sr ratio (cm)	43.2 ± 5.4	57.8 ± 4.8^∗^	92.1 ± 25.1^∗^^†^	<0.001
GLS (%)	−18.4 ± 3.9	−17.2 ± 3.7	−14.9 ± 3.9^∗^^†^	<0.001

ABI: ankle-brachial index; baPWV: brachial-ankle pulse wave velocity; AoAC: aortic arch calcification score; hs-CRP: high-sensitivity C-reactive protein; ACE: angiotensin-converting enzyme; ARB: angiotensin II receptor blocker; LAVI: left atrial volume index; LVMI: left ventricular mass index; LVEF: left ventricular ejection fraction; E: peak early transmitral filling wave velocity; E': early diastolic velocity of lateral mitral annulus; E'sr: global diastolic strain rate; GLS: global left ventricular longitudinal systolic strain. ^∗^*p* < 0.05 compared with the 1^st^ tertile of the E/E'sr ratio; ^†^*p* < 0.05 compared with the 2^nd^ tertile of the E/E'sr ratio.

**Table 2:** Comparison of baseline characteristics between patients with preserved GLS (≤−16%) or impaired GLS (>−16%).

Characteristics	Preserved GLS (≤−16%) (*n* = 110)	Impaired GLS (>−16%) (*n* = 80)	*p*
Age (year)	60.4 ± 11.8	61.4 ± 11.8	0.565
Male gender (%)	48.2	56.3	0.272
Dialysis vintage (year)	6.9 (2.6–12.5)	6.4 (2.5–10.2)	0.266
Diabetes mellitus (%)	34.5	61.3	<0.001
Coronary artery disease (%)	7.3	23.8	0.001
Cerebrovascular disease (%)	5.5	15.0	0.027
Body mass index (kg/m^2^)	23.3 ± 3.4	24.2 ± 4.4	0.133
Systolic blood pressure (mmHg)	151.5 ± 27.4	160.8.4 ± 27.3	0.032
Heart rate (beat/min)	76.9 ± 11.2	80.1 ± 11.1	0.055
ABI < 0.95 (%)	24.5	43.8	0.039
baPWV (cm/s)	1876.3 ± 499.6	1983.9 ± 583.5	0.207
Cardiothoracic ratio (%)	49.6 ± 6.3	49.9 ± 6.1	0.744
AoAC	2 (0–5)	3 (0–7)	0.022
Laboratory parameters			
Albumin (g/dL)	3.8 ± 0.3	3.9 ± 0.3	0.584
Triglycerides (mg/dL)	115.5 (88.8–211)	136.5 (82–199.8)	0.942
Total cholesterol (mg/dL)	178.1 ± 41.1	177.7 ± 39.1	0.938
Hemoglobin (g/dL)	10.3 ± 1.1	10.7 ± 1.3	0.028
Creatinine (mg/dL)	9.7 ± 2.4	9.4 ± 2.2	0.404
Calcium-phosphorous product (mg^2^/dL^2^)	40.1 ± 11.6	44.2 ± 11.2	0.016
hs-CRP (mg/L)	2.2 (0.9–5.4)	3.1 (1.2–8.6)	0.227
Medications			
ACE inhibitors or ARBs (%)	19.1	26.3	0.240
*β*-Blockers (%)	20.0	23.8	0.535
Statins (%)	20.0	25.0	0.412
Echocardiographic data			
LAVI (mL/m^2^)	34.6 ± 12.5	31.8 ± 10.3	0.113
LVMI (g/m^2^)	129.9 ± 40.5	143.8 ± 43.7	0.026
LVEF (%)	69.9 ± 7.6	62.2 ± 11.2	<0.001
Relative wall thickness	0.42 ± 0.09	0.42 ± 0.11	0.788
E/E' ratio	13.3 ± 7.7	15.9 ± 11.4	0.077
E/A ratio	0.89 ± 0.29	0.82 ± 0.38	0.155
Deceleration time (ms)	190.0 ± 61.2	184.7 ± 62.9	0.788
E' wave (cm/s)	7.4 ± 2.2	5.7 ± 2.0	<0.001
E'sr (1/s)	1.55 ± 0.35	1.08 ± 0.24	<0.001
E/E'sr ratio (cm)	58.3 ± 21.3	73.0 ± 28.3	<0.001
GLS (%)	−19.6 ± 2.4	−13.1 ± 2.6	<0.001

ABI: ankle-brachial index; baPWV: brachial-ankle pulse wave velocity; AoAC: aortic arch calcification score; hs-CRP: high-sensitivity C-reactive protein; ACE: angiotensin-converting enzyme; ARB: angiotensin II receptor blocker; LAVI: left atrial volume index; LVMI: left ventricular mass index; LVEF: left ventricular ejection fraction; E: peak early transmitral filling wave velocity; E': early diastolic velocity of lateral mitral annulus; E'sr: global diastolic strain rate; GLS: global left ventricular longitudinal systolic strain.

**Table 3:** Associations of the E/E'sr ratio and GLS with overall mortality, CV mortality, and MACE using the Cox proportional hazards model.

	Overall mortality		CV mortality		MACE	
	HR (95% CI)	*p*	HR (95% CI)	*p*	HR (95% CI)	*p*
E/E'sr ratio (per 10 cm)						
Crude	1.191 (1.090–1.301)	<0.001	1.271 (1.135–1.423)	<0.001	1.188 (1.101–1.282)	<0.001
Age and sex adjusted	1.185 (1.074–1.308)	0.001	1.261 (1.112–1.429)	<0.001	1.175 (1.083–1.274)	<0.001
Model 1 adjusted	1.392 (1.147–1.690)	0.001	1.476 (1.068–2.040)	0.018	1.188 (1.029–1.372)	0.019
Model 2 adjusted	1.484 (1.201–1.834)	<0.001	1.584 (1.058–2.371)	0.025	1.205 (1.040–1.397)	0.013
GLS (per 1%)						
Crude	1.127 (1.043–1.219)	0.003	1.202 (1.075–1.343)	0.001	1.174 (1.100–1.254)	<0.001
Age and sex adjusted	1.139 (1.046–1.239)	0.003	1.208 (1.072–1.360)	0.002	1.173 (1.095–1.255)	<0.001
Model 1 adjusted	1.266 (1.100–1.457)	0.001	1.312 (1.055–1.632)	0.015	1.212 (1.098–1.337)	<0.001
Model 2 adjusted	1.276 (1.101–1.480)	0.001	1.513 (1.088–2.104)	0.014	1.214 (1.103–1.337)	<0.001

CV: cardiovascular; MACE: major adverse cardiovascular events. Model 1 comprises age, sex, dialysis vintage, diabetes mellitus, coronary artery disease, cerebrovascular disease, body mass index, systolic blood pressure, ABI < 0.95, baPWV, cardiothoracic ratio, AoAC, albumin, triglycerides, total cholesterol, hemoglobin, creatinine, calcium-phosphorous product, and hs-CRP. Model 2 comprises model 1 plus the use of ACE inhibitors or ARBs, beta-blockers, and statins.

**Table 4:** Comparisons of the E/E' ratio with the E/E'sr ratio and LVEF with GLS in the prediction of overall mortality, CV mortality, and MACE.

	Overall mortality		CV mortality		MACE	
	*χ* ^2^ change	*p*	*χ* ^2^ change	*p*	*χ* ^2^ change	*p*
Basic model+E/E' ratio	0.509	0.476	0.005	0.943	0.422	0.516
Basic model+E/E'sr ratio	13.914	<0.001	6.833	0.009	5.424	0.020
Basic model+LVEF	0.484	0.487	1.234	0.267	1.167	0.280
Basic model+GLS	12.007	0.001	10.189	0.001	15.682	<0.001

*p* value was based on the incremental value compared with the basic model adjusted for age, sex, dialysis vintage, diabetes mellitus, coronary artery disease, cerebrovascular disease, body mass index, systolic blood pressure, ABI < 0.95, baPWV, cardiothoracic ratio, AoAC, albumin, triglycerides, total cholesterol, hemoglobin, creatinine, calcium-phosphorous product, hs-CRP, and the use of ACE inhibitors or ARBs, beta-blockers, and statins.

## Data Availability

The data supporting the findings of this study are available within the article or are available from the corresponding author upon reasonable request.
